# Cross-cultural adaptation and validation to Brazilian Portuguese of the ‘knowledge of gestational diabetes (GDM)’ questionnaire for women with GDM

**DOI:** 10.1186/s13098-024-01456-z

**Published:** 2024-09-14

**Authors:** Laura Betat Pereira, Helena Trevisan Schroeder, Juciela Keller dos Santos, Paulo César Brizolla Capelari, Beatriz D. Schaan, Patrícia Martins Bock

**Affiliations:** 1 Post-Graduate Program in Pharmacology and Therapeutics, Universidade Federal do Rio Grande do Sul, Ramiro Barcelos, 2600, Porto Alegre, RS 90035-003 Brasil; 2 Post-Graduate Program in Physiology, Universidade Federal do Rio Grande do Sul, Ramiro Barcelos, 2600, Porto Alegre, RS 90035-003 Brasil; 3 Nursing Department, Universidade La Salle, Victor Barreto, 2288, Canoas, RS 92010-000 Brasil; 4 Nursing Department, Anhanguera Educacional, Marechal Floriano Peixoto, 142, Porto Alegre, RS 90020-060 Brasil; 5 Internal Medicine Department, Medical School and Post-Graduate Program in Medical Sciences: Endocrinology, Universidade Federal do Rio Grande do Sul, Ramiro Barcelos, 2400, Porto Alegre, RS 90035-903 Brazil; 6 Endocrine Division, Hospital de Clínicas de Porto Alegre, Rua Ramiro Barcelos 2350, Porto Alegre, RS Brazil; 7 Universidade Federal do Rio Grande, Km 8 Avenida Itália Carreiros, Rio Grande, RS 96203-90 Brazil

**Keywords:** Diabetes mellitus, Gestational, Psychometrics, Health knowledge, Attitudes, Practice, Gestational diabetes, Validation

## Abstract

**Background:**

Gestational diabetes mellitus (GDM) is characterized by hyperglycemia during pregnancy. There are many diabetes-specific tools for collecting information validated in Brazilian Portuguese. However, there are no specific instruments to assess knowledge about GDM in Brazilian Portuguese. The aim of this study was to cross-culturally adapt and validate the Brazilian Portuguese version of the Knowledge of Gestational Diabetes Mellitus questionnaire (GDMKQ).

**Methods:**

This study was conducted in southern Brazil from January to December 2023. Women with GDM or diabetes identified during pregnancy were considered eligible based on specific criteria. Clinical and demographic data were obtained through a medical records search. The GDMKQ underwent a multistep adaptation process, including translation, back-translation, content validity assessment, and cognitive interviews. After administration to participants, internal consistency, item-total correlation, and intraclass correlation were assessed. Confirmatory factor analysis was also conducted to ensure validity.

**Results:**

A total of 155 pregnant women were recruited for the study. Most participants were aged 18–30 years, and hypertension was the main comorbidity (25.2%). Regarding education, most participants (58.7%) attended high school. The Brazilian Portuguese version of the GDMKQ consisted of 32 items. The intraclass correlation was established by two independent interviews with 57 participants, yielding a correlation coefficient of 0.79 (p < 0.01). Internal consistency (Cronbach’s alpha) was 0.81 overall but was less than 0.7 for each domain. Item-total correlations were calculated, and confirmatory factor analysis indicated a good model fit. The final Brazilian Portuguese version of the questionnaire consisted of 32 items.

**Conclusions:**

The Brazilian Portuguese version of the GDMKQ yielded a reliable and valid tool for evaluating diabetes knowledge in pregnant women.

**Supplementary Information:**

The online version contains supplementary material available at 10.1186/s13098-024-01456-z.

## Background

Gestational diabetes mellitus (GDM) is diabetes mellitus diagnosed in the second or third trimester of pregnancy that is not clearly overt pre-pregnancy diabetes or other types of diabetes that occur during pregnancy, such as type 1 diabetes [1]. The International Diabetes Federation 2021 estimates that hyperglycemia during pregnancy affects approximately one in six pregnancies [2]. In Brazil, the prevalence of GDM is approximately 18% [3].

Gestational diabetes mellitus can cause significant complications for both mother and baby, including gestational hypertension, pre-eclampsia, and an increased risk of type 2 diabetes for the mother, and macrosomia and increased risk of obesity and diabetes later in life for the baby. Effective management of GDM depends on adequate knowledge, to enable pregnant women to control blood glucose levels and adopt a healthy lifestyle. Well-designed educational programs are essential to empower women to manage their condition and improve both maternal and neonatal outcomes [4, 5].

Studies assessing women’s knowledge and awareness of GDM have shown a high prevalence of poor awareness and knowledge [6, 7]. In addition, educated women are more likely to be aware of the importance of lifestyle and self-management, and women with higher incomes may be able to afford better nutritional and medical care [8]. Thus, knowledge may play an important role in improving GDM control. The use of tools such as questionnaires could facilitate the early identification of poor knowledge and the implementation of preventive measures.

There are more than 20 diabetes-specific instruments validated in Brazilian Portuguese, all of which are important tools for collecting information about people’s behavior, knowledge, and attitudes related to diabetes through the administration of standardized questions. However, there is no specific instrument for the assessment of GDM [9]. Therefore, the GDMKQ was found to be appropriate for this purpose. The GDMKQ, originally validated by Carolan-Olaha and Vasilevski in Australia, is designed to assess three domains: understanding of GDM, including normal blood glucose levels and its impact on both mother and baby; knowledge of nutritional values and food choices; and knowledge of GDM self-management principles, including blood glucose monitoring, exercise, and dietary habits [10].

The assessment of GDM knowledge and treatment adherence is challenging, mainly due to the lack of validated instruments for this purpose in women with GDM in Brazil. The use of questionnaires for this purpose, due to their ease of use and low cost, could enable healthcare teams to develop patient education interventions and provide a practical and rapid method for use in clinical settings as well, as in healthcare research.

## Methods

The aim of this study was to cross-culturally adapt and validate the Brazilian Portuguese version of the (GDMKQ). This study was conducted in a high-risk pregnancy outpatient clinic of two university hospitals in southern Brazil (Hospital Materno Infantil Presidente Vargas, HMIPV and Hospital de Clínicas de Porto Alegre, HCPA) from January to December 2023, in accordance with the Declaration of Helsinki. This study was approved by the Scientific Committee and Research Ethics Committee of HCPA (Brazil) (Certificate of Presentation for Ethical Appreciation 62984522.9.1001.5327) and the Scientific Committee and Research Ethics Committee of HMIPV (Certificate of Presentation for Ethical Appreciation 62984522.9.2001.5329). Pregnant women with a diagnosis of GDM or with diabetes diagnosed during pregnancy were selected from the medical record database of the high-risk antenatal clinics at both hospitals. Those who met the eligibility criteria were invited to participate in the study and gave their consent by signing the informed consent form.

### Participants

Pregnant women were considered eligible if they were 18 years of age or older, had a diagnosis of diabetes during pregnancy or had been diagnosed with GDM at least 1 month before, identified with a Brazilian cultural identity, and had consulted with at least one healthcare professional. For the diagnosis of GDM, the criteria were fasting blood glucose levels between 92 and 125 mg/dL, 1-h plasma glucose levels of 180 mg/dL or higher, or 2-h plasma glucose levels between 153 and 199 mg/dL. For overt diabetes, the criteria were a fasting glucose levels greater than 126 mg/dL or a random blood glucose level greater than 200 mg/dL [1]. Exclusion criteria included known pre-pregnancy diabetes, previous GDM, pregnant women without blood glucose records, and any communication or comprehension barriers, such as mental disorders or illiteracy. Interested eligible patients signed an informed consent form. Inclusion and exclusion criteria were defined by the investigators LBP, BDS and PMB.

Clinical and demographic data, including the women’s age, place of residence, level of education, current gestational age, self-monitoring of capillary glucose results, insulin use, current medications, diagnostic test results (transcribed if performed elsewhere or in the hospital), and family history of diabetes were obtained from the patient’s medical records.

### Instrument

The authors of the original questionnaire were asked for permission by email, and the study began only after their approval. The GDMKQ, which was originally validated by Carolan-Olah and Vasilevsli in 2021, is a structured interview designed to assess pregnant women’s knowledge of GDM (Additional file 1). The questionnaire consists of 33 questions covering the following domains: 1. Knowledge of GDM, including normal blood glucose levels and the effects of GDM on mothers and babies; 2. Knowledge of nutritional values and food choices; 3. Knowledge of principles of GDM self-management, including blood glucose monitoring, physical activity, and dietary habits. Answers are numerically coded, and each correct answer is scored as 1 point. Most questions have one correct answer and are scored as either true or false. Four questions have more than one correct answer and are scored as correct (all correct answers identified) or incorrect (all correct answers not identified) [10].

### Procedures and measures

The GDMKQ underwent a multi-step process, including translation and content validity assessment. After administration to participants, internal consistency, item-total correlation, and intraclass correlation were assessed. Confirmatory factor analysis was also used to ensure validity.

The initial translation of the original instrument into Brazilian Portuguese was done by two independent translators who were native speakers of Brazilian Portuguese, fluent in both languages, and from different professional backgrounds than the researchers. In the synthesis process, a consensus was reached from the resulting versions, and words and phrases that showed differences were readapted [11]. Content validity was assessed by experts in the field and by the target audience.

A committee of experts in the field was assembled to produce a final version of the modified instrument. This committee included a Pharm.D. pharmacist, a Ph.D. pharmacist and a Ph.D. endocrinologist, all of whom are bilingual [12]. The committee carefully reviewed each item of the instruments to better adapt them, ensure that the translation was fully comprehensible, and verify cross-cultural equivalence between the source and final versions. Semantic, idiomatic, empirical, and conceptual aspects were considered [12]. The synthetic version of the instrument was back-translated by a translator who is a native speaker of the source language and fluent in the target language. This translator had no prior knowledge of the instrument. Based on the translation and back-translation, a final version of the instrument was obtained [13].

The final stage of the adaptation process was the assessment of content validity by the target population, also known as cognitive debriefing or pretesting, in which the instrument was administered to 30 women diagnosed with GDM or with diabetes detected during pregnancy. A sample size of 30 to 40 individuals is recommended for pretesting in questionnaire validation and cultural adaptations studies [11, 13]. Participants completed the questionnaire and were interviewed to assess the comprehensibility, interpretability, and cultural relevance of the translation. Cognitive debriefing was used to obtain feedback from participants and confirm the acceptability of the translation [11–13]. The content validity coefficient (CVC) was determined by calculating the items related to the clarity, appropriateness, and comprehensibility of the questions, which were rated on a scale of 1 to 5. A CVC ≥ 0.70 for each item and for the instrument as a whole was used as the cut-off point to determine satisfactory levels of language clarity and appropriateness [14]. The instrument was administered to pregnant women at least 4 weeks after their diagnosis and with an expected delivery date of at least 1 month. Participants’ privacy was protected during the interviews.

Upon completion of this cross-cultural adaptation, and based on a suggestion regarding validation sample size [15], a further 125 patients were selected to complete the final version in order to determine the reliability and validity of the instrument. Intraclass correlation was established by two independent interviews of 57 participants within 1–12 weeks of the previous assessment. The number of second interviews was adequate according to previous instrument validation studies [16, 17].

### Statistical analysis

The Statistical Package for Social Science Professional program version 20.0 (IBM Corp., Armonk, NY, USA) and the R package lavaan (version 0.6–17) were used to perform the analyses.

Interrater agreement was measured using the intraclass correlation coefficient. Intraclass correlation values below 0.5 indicate poor reliability, values between 0.5 and 0.75 indicate moderate reliability, values between 0.75 and 0.9 indicate good reliability, and values above 0.90 indicate excellent reliability [18]. Cronbach’s alpha (α) was calculated to measure internal consistency, and values above 0.7 were considered acceptable [12]. In addition, the effect of removing each question on the Cronbach’s alpha value, which may result in the exclusion of a particular item after confirmatory factor analysis, was examined. Item-total correlations were calculated. The inclusion of items with values above 0.2 is considered appropriate, and negative values, indicating a negative correlation, may represent a question that is inversely proportional to the others [19, 20]. The confirmatory factor analysis model was fitted using diagonal weighted least squares with robust standard errors. The comparative fit index (CFI) was used to analyze the model fit by examining the discrepancy between the data and the hypothesized model, taking into account sample size issues inherent in the chi-squared test and the normed fit index [21].

## Results

**Fig. 1:**
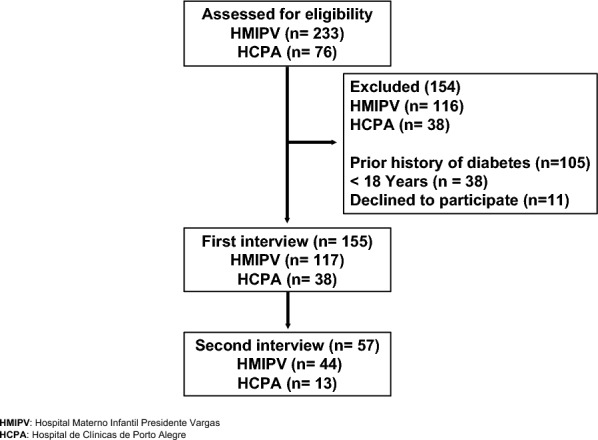
Flow diagram

Of 309 pregnant women screened for eligibility, 155 were recruited for the study. The instrument was administered, and analyses were conducted with 155 participants, while cognitive debriefing was conducted with 30 participants. Fifty-seven participants answered the same questions with a second interviewer. Figure 1 shows the flow chart for the selection of participants in each stage of the study.

### Sociodemographic and clinical characteristics

Most participants were aged 18–30 years, and the median age was 29 (24–35) years. Gestational age at the interview was greater than 25 weeks in 131 pregnant women (84.5%) and the diagnosis of GDM was made before the end of the second trimester of pregnancy (up to 24 weeks) in 98 participants (63.9%). Ninety pregnant women were from the state capital (58.1%). Regarding the education of participants, most (58.7%) had completed high school. Hypertension was the most common comorbidity and was diagnosed in about a quarter of the women (25.2%). Most pregnant women were taking vitamin supplements (54.2%), and 39.4% were taking insulin. The sociodemographic and clinical characteristics of the patients are shown in Table 1.

**Table 1:** Demographic and clinical characteristics of participants

Characteristics (n = 155)		n (%)	IC 95%
Age (years)	18–25	42 (27.1)	20.7–34.6
	26–30	42 (27.1)	20.7–34.6
	31–35	33 (21.3)	15.6–28.4
	> 36	38 (24.5)	18.4–31.9
Gestational age at interview (weeks)	1–12	1 (0.6)	0.0–1.0
	13–24	23 (14.8)	14.0–33.0
	≥ 25	131 (84.5)	122.0–141.0
Gestational age at GDM diagnosis (weeks)	1–12	72 (46.5)	60.0–85.0
	13–24	27 (17.4)	18.0–37.0
	≥ 25	10 (6.5)	4.0–17.0
	Uniformed	46 (29.7)	35.0–58.0
Instruction	Elementary school	35 (22.6)	16.7–29.8
	High school	91 (58.7)	50.8–66.2
	Higher education	23 (14.8)	10.1–21.3
	Uninformed	6 (3.9)	1.8–8.2
Comorbidity	Hypertension	39 (25.2)	19.0–32.5
	Obesity	21 (13.5)	9.0–19.8
	Hypothyroidism	18 (11.6)	7.5–17.6
	Nervous system diseases^1^	25 (16.1)	11.2–22.7
	Infectious diseases^2^	11 (7.1)	4.0–12.3
	Other^3^	20 (12.9)	8.5–19.1
Medications	Insulin	61 (39.4)	32.0–47.2
	Methyldopa	34 (21.9)	16.1–29.1
	Prevention of pre-eclampsia^4^	42 (27.1)	20.7–34.6
	Supplements and vitamins^5^	84 (54.2)	46.3–61.8
	Nervous system medication^6^	20 (12.9)	8.5–19.1
	Metformin	13 (8.4)	5.0–13.8
	Other^6^	35 (22.6)	16.7–29.8
Reported self-monitoring	Yes	138 (89.0)	130.0–147.0
	No	17 (11.0)	9.0–26.0
Blood glucose (mg/dL)	Fasting	95 ± 13	93–97
	1 h after meal	113 ± 18	110–116

Categorical variables are expressed as absolute and relative frequencies. Continuous variables are expressed as mean ± standard deviation

^1^Migraine, anxiety, depression, epilepsy, borderline

^2^Syphilis, toxoplasmosis, and hepatitis

^3^Cardiovascular system. Coagulation disorders, neoplasias, respiratory disease

^4^Acetyl salicylic acid and calcium

^5^Ferrous sulfate, folinic acid and vitamin D

^6^Sertraline, escitalopram, fluoxetine, carbamazepine, quetiapine; ^7^Sulfadiazine and pyridoxine, levothyroxine. GDM: Gestational diabetes mellitus

Sixty-two pregnant women (40%) had a family history of diabetes, while 57 (36.8%) did not have this information in their medical records. Regarding self-monitoring of blood glucose, 138 subjects (89.0%) performed at least two capillary blood glucose tests in the week prior to the interview. They reported mean blood glucose levels of 95 ± 13 mg/dL and 113 ± 18 mg/dL when fasting and 1 h after a meal, respectively. Glycated hemoglobin (HbA1c) was reported in the medical records of 47 patients (30.3%), with a mean value of 5.2% ± 0.4.

### Cross-cultural adaptation of the instrument

Content validity of the original GDMKQ by the expert evaluation committee revealed discrepancies between the versions provided by the two original translators in 13 of the 33 questions, and a consensus was reached. In addition, the other four questions were modified for cross-cultural equivalence (Supplementary Table 1). The result of the back-translation remained faithful to the original document.

Content validity was assessed by the target group (cognitive debriefing) with 30 pregnant women. The comprehensibility, interpretation, and cultural relevance of the translation confirmed the acceptability of the translation, as most women had no difficulty in understanding any of the questions. Only five women expressed mild difficulty with 7 questions. The CVC for each question was 1 for 26 questions, 0.99 for 6 questions, and 0.98 for 1 question. After scoring the responses, the final CVC value was 0.997.

### Reliability and validity

The Brazilian Portuguese version of the GDMKQ instrument consisted of 33 items and was completed in approximately 20 min. The percentage of correct responses for each question is described in Table 2. Most questions had one correct answer and were scored as either true or false. Four questions had more than one correct answer (questions 1, 2, 3 and 5) and were scored as correct (all correct answers identified) or incorrect (all correct answers not identified). The questions with multiple correct answers had the highest proportion of incorrect answers.

**Table 2:** Percentages of correct scores (n = 155) for questions in each of the domains of the Knowledge of Gestational Diabetes Questionnaire

Domain 1: knowledge of GDM	Correct (%)	Domain 2: knowledge of nutrition values	Correct (%)	Domain 3: knowledge of GDM self-management	Correct (%)
Q1	3.9	**Q15**	63.2	**Q20**	88.4
Q2	0	**Q16**	84.5	**Q21**	84.5
Q3	9.7	**Q17**	59.4	**Q22**	28.4
Q4	91	**Q18**	87.7	**Q23**	52.3
Q5	11	**Q19**	50.3	**Q24**	56.8
Q6	67.7			**Q25**	83.2
Q7	80			**Q26**	65.2
Q8	84.5			**Q27**	98.1
Q9	77.4			**Q28**	81.9
Q10	58.1			**Q29**	57.4
Q11	67.7			**Q30**	70.3
Q12	61.9			**Q31**	73.5
Q13	81.9			**Q32**	92.9
Q14	69.7			**Q33**	50.3

The intraclass correlation coefficient was 0.79 (p < 0.01), indicating good reliability. The internal consistency (Cronbach’s alpha) found in our study was 0.81; however, the internal consistency by domain was < 0.7 (Table 3). Item-total correlations were calculated separately for each item, and except for items 2 and 14, they were higher than the recommended level and correlated very well with the overall scale (Table 4). In terms of internal consistency reliability analysis, most item-total correlations were positive and greater than 0.2. One item returned a negative value (question 14), indicating a negative correlation. Confirmatory factor analysis was conducted to examine an “a priori” three-factor structure consisting of each domain identified as a factor. Item 2 was removed, reducing the original 33-item scale to 32 items. These changes were made in one set and resulted in improved model fit. The comparative fit index value of 0.936 and the root mean square error of approximation of 0.048 (95% confidence interval 0.038–0.057) are accepted as indicators of good fit.

**Table 3:** Internal consistency by domains

Knowledge domain (max score)	Internal consistency (Chronbach’s α) (n = 155)
1. Knowledge of GDM	0.55
2. Knowledge of nutrition values	0.55
3. Knowledge of GDM self-management	0.72

*GDM* Gestational diabetes mellitus

**Table 4:** Item-total correlation

Question	Scale mean if item deleted	Scale variance if item deleted	Corrected item-total correlation	Cronbach’s alpha if item deleted
1	20.8903	25.306	0.164	0.811
2	20.9290	25.664	0.000	0.812
3	20.8323	24.959	0.208	0.810
4	20.0194	24.798	0.273	0.808
5	20.8194	24.798	0.246	0.809
6	20.2516	23.462	0.436	0.801
7	20.1290	24.620	0.222	0.810
8	20.0839	24.740	0.219	0.809
9	20.1548	24.872	0.147	0.812
10	20.3484	24.034	0.285	0.808
11	20.2516	23.969	0.321	0.806
12	20.3097	23.033	0.512	0.798
13	20.1097	24.670	0.220	0.810
14	20.2323	25.829	-0.081	0.822
15	20.2968	23.613	0.387	0.803
16	20.0839	24.558	0.271	0.808
17	20.3355	23.705	0.358	0.805
18	20.0516	24.621	0.286	0.807
19	20.4258	23.038	0.493	0.798
20	20.0452	24.290	0.401	0.804
21	20.0839	24.207	0.371	0.805
22	20.6452	24.750	0.158	0.813
23	20.4065	22.983	0.506	0.798
24	20.3613	22.960	0.516	0.797
25	20.0968	23.478	0.563	0.798
26	20.2774	23.474	0.423	0.802
27	19.9484	25.478	0.120	0.811
28	20.1097	24.683	0.217	0.810
29	20.3548	23.841	0.326	0.806
30	20.2258	24.384	0.236	0.809
31	20.1935	23.378	0.488	0.799
32	20.0000	24.636	0.376	0.806
33	20.4258	24.220	0.241	0.810

Seventy-three women (47.1%) achieved the fasting glycemic target, taking into account capillary glucose, in the measurements taken in the week prior to the interview. Seventy-nine women (51%) did not reach the target suggested by the Brazilian Diabetes Society (95 mg/dL), and three women (1.9%) did not have their measurements documented in their medical records.

The final Brazilian Portuguese version of the (GDMKQ) consisted of 32 items and is presented in Additional file 2.

## Discussion

The results of this study support the validity of a Brazilian Portuguese version of the GDMKQ questionnaire for women with GDM, indicating that it could be reliably used in future studies in Brazil.

The reliability of the instrument was assessed using several methods. The internal consistency (Cronbach’s alpha) found in our study was 0.81, which is adequate [22], suggesting that despite the modifications, the scale remained internally consistent despite the modifications. However, the Cronbach’s alpha of the GDMKQ domains, when analyzed separadely, was < 0.7 for each domain, indicating the unreliability of the subscales when used separately. A decrease in reliability when domains are analyzed separately has also been observed in other studies [16].

Most questions in the original instrument have one correct answer and are scored as either correct or incorrect. Four questions had more than one correct answer and were scored as correct (all correct answers identified) or incorrect (all correct answers not identified). Since partially correct responses were treated as incorrect in the scoring process, this criterion resulted in a significant number of responses being scored as incorrect in the questions with more than one correct answer, leading to a notably high percentage of inaccuracies. Despite the high prevalence of incorrect responses, these questions were retained for analysis. Possible solutions could include considering a partially correct answer as acceptable or reformulating the questions to ensure that they offer only one correct option. Question 22, on exercise intensity, also had a high percentage of incorrect responses, despite having only one correct option. One possible explanation is that this is a physical activity question, and pregnant women often lack professional guidance on the appropriate intensity of exercise. As a result, there is confusion about what constitutes “light” (“leve”) or “moderate” (“moderado”) exercise [23].

In the item-total correlation analysis, alpha values did not change significantly after excluding a particular item, with the satisfactory alpha value remaining unchanged. Question 2, with had no recorded correct answers, was not included in the confirmatory factor analysis. After the confirmatory factor analysis, the test indicated that the model was well fitted. In addition, the two independent raters agreed on the total scores.

The results of internal reliability, item-total correlation, and intraclass correlation are consistent with studies that have also validated instruments [24–26], despite the difficulty in finding studies assessing knowledge about GDM. This fact reinforces the importance of noninvasive assessment tools that target the respondent population. However, the original questionnaire does not provide a score to determine good knowledge. Therefore, our study also did not categorize the scores obtained.

Given the extensive and multifaceted consequences of a diagnosis of GDM, it is challenging to assess adequate control using only a single capillary glucose measurement. Continuous glucose monitoring, a new method addressed in some studies, offers an alternative for accurate assessment of glycemic control. This method provides more accurate values and has shown promising results [27, 28]. We recognize that analysis of the relationship between scale scores and glycemic control (predictive validity) should be useful; however, we cannot accurately assess glycemic control.

Several international reports have attempted to assess pregnant women’s knowledge of GDM [7, 29–38]. Although many of these instruments are not standardized or have not been fully validated, they have shown that low educational level, maternal age, history of GDM or hypertension and number of pregnancies may be associated with understanding and managing GDM [34–38]. To our knowledge this is the first article to cross-culturally adapt and validate a version of the GDMKQ in another language after its validation. This reiterates once again the importance of this work to generate a validated tool, in this case specifically for the Brazilian sample, and reinforces the possibility of using the questionnaire developed by Carolan-Olaha and Vasilevski and its validation in other populations [10]. In addition, it is important to note that the original article from which the questionnaire was developed is relatively recent, and there may not have been enough time to conduct and publish a study. There are several limitations to consider in this study. First, the test–retest evaluation period ranged from 1 to 12 weeks, which is consistent with the typical frequency of multidisciplinary consultations for pregnant women. The extended interval was due to women missing appointments or not having time to complete the questionnaire. This time frame may explain the increased learning and discrepancies in responses between the two interviews. In addition, this period coincided with various physiological changes in the participants’ bodies, concerns about the baby, and the need to adjust to numerous new situations, all of which may have affected their willingness to participate in the study. In this population, the onset of diabetes is only one aspect of the many changes experienced during pregnancy.

Overall, the translation, adaptation, and validation processes indicated that the GDMKQ accurately assessed knowledge about diabetes in pregnant women. Therefore, the adapted Brazilian Portuguese version of the evaluated instrument showed satisfactory psychometric properties and validity, producing comparable results, except for its specific characteristics. It serves as an important alternative for researchers to assess and understand pregnant women’s awareness of GDM. The tool effectively identifies knowledge gaps among pregnant women with diabetes and has the potential to improve the effectiveness of educational strategies and interventions aimed at supporting diabetes self-care.

## Conclusions

The results of this study provide substantial evidence for the validity of a Brazilian Portuguese version of an instrument designed to assess knowledge about GDM. This suggests that the instrument holds promise for reliable use in future research efforts.

## Supplementary Information

Additional file 1.

Additional file 2.

Additional file 3.

## Data Availability

The datasets generated and analyzed during the current study are not publicly available, but are available from the corresponding author.

## Abbreviations

CFI: Comparative fit index
CVC: Content validity coefficient
GDM: Gestational diabetes mellitus
HCPA: Hospital de Clínicas de Porto Alegre
HMIPV: Hospital Materno Infantil Presidente Vargas
