# Supplementary material for: Cross-cultural adaptation and validation to Brazilian Portuguese of the ‘knowledge of gestational diabetes (GDM)’ questionnaire for women with GDM
Source: Diabetol Metab Syndr. 2024 Sep 14;16:227. doi: 10.1186/s13098-024-01456-z (PMC11401380; doi:10.1186/s13098-024-01456-z)
Supplement: Supplementary file 1 — Additional file 1. [file 13098_2024_1456_MOESM1_ESM.pdf]

Gestational Diabetes Mellitus (GDM) Questionnaire

The majority of questions have one correct answer and are scored as either correct or incorrect. Four questions (question 1, 2, 3 and 5) have more than one correct answer, and are scored as correct (all correct answers identified) or incorrect (did not identify all correct answers).

Knowledge of Gestational Diabetes

Below are some statements about diabetes. There may be more than one correct answer. After reading the statement please circle whatever answers you believe are true. If you do not know the answer please circle a number (I don't know).

These questions may have more than <u>one</u> correct answer

1. Because I have gestational diabetes, my baby may be:

a) larger than usual
b) smaller than usual
c) born early
d) admitted to special care
e) I don't know

2. Women are more likely to develop gestational diabetes if they:

a) are overweight
b) have had more than 3 children
c) are over 30 years
d) are from India, Asia or the Middle east
e) I don't know

3. Because I have gestational diabetes, I may:

a) need to come to the clinic more frequently
b) need a caesarean section
c) develop permanent diabetes later in life
d) I don't know

4. In uncontrolled diabetes the blood sugar is:

a) normal
b) increased
c) decreased
d) I don't know

5. Gestational diabetes is:

a) present during pregnancy
b) disappears once the baby is born
c) may lead to diabetes in later life

d) is not very serious
e) I don't know

5. **Gestational diabetes may be treated with:**

a) diet
b) diet and exercise
c) insulin
d) All of the above
e) I don't know

6. **When my baby is born:**

a) My diabetes will disappear
b) I don't need to worry about being diabetic any more
c) I should get a follow up glucose test at my 6 weeks check up
d) I don't know

Knowledge on testing blood glucose level

The following questions require you to circle **ONE** number only.

7. **A normal fasting (on an empty stomach) blood glucose level is:**

a) less than 5mmol/L
b) less than 6mmol/L
c) 7mmol/L or more
d) 8mmol/L or more
e) I don't know

8. **A normal 2 hour blood glucose level is:**

a) less than 5mmol/L
b) less than 6.7mmol/L
c) 7mmol/L or more
d) 8mmol/L or more
e) I don't know

9. **I should test my blood glucose level:**

a) in the morning before breakfast
b) in the afternoon before lunch
c) 2 hours after meals
d) both 1 and 3
e) I don't know

10. **What do I do if my blood glucose level is high on one occasion?**

a) make a note in your diary
b) check what you ate before the high blood glucose level
c) both 1 and 2
d) I don't know

11. **What do I do if my blood glucose level is high on two occasions in one week?**

a) make a note in your diary
b) check what you ate before the high blood glucose level
c) contact the diabetes educator

d) all of the above
e) I don’t know

12. Should I take my blood glucose level if I am feeling sick and haven’t eaten?

a) yes, continue to take your blood glucose levels as usual
b) no, do not take your blood glucose levels until you are feeling better
c) I don’t know

13. When you prick your finger, you should:

a) use the same finger every day
b) use a different finger every day
c) it is not important
d) I don’t know

Knowledge of Food choice and looking after yourself after GDM diagnosis
(Please choose <u>ONE</u> only)

14. The preferred type of carbohydrate foods are:

a) white bread
b) wholemeal bread
c) wholegrain foods that are high in fiber
d) foods high in starch
e) I don’t know

15. What form of fruits and vegetables are better?

a) fruit or vegetable juices
b) processed or tinned fruits and vegetables
c) fruits with added fats, sugar and salt
d) fresh fruit and vegetables
e) I don’t know

16. Protein intake can be obtained from:

a) meat
b) fish
c) nuts
d) dairy such as milk or cheese
e) all of the above
f) I don’t know

17. What type of protein is best?

a) skinless baked chicken or turkey
b) skin-on chicken or turkey
c) deep fried or stir fried chicken or turkey
d) any chicken or turkey
e) I don’t know

18. A balanced diet should have:

a) more vegetables
b) less carbohydrates such as white bread
c) low fat and low sugar choices

d) all of the above
e) I don't know

19. Exercise in GDM helps to:

a) controls mother's blood glucose and improves baby's health
b) is not helpful
c) tires you out
d) does none of the above
e) I don't know

20. Exercises that are recommended during pregnancy are:

a) netball, basketball and hockey
b) running and skipping
c) walking, swimming and yoga
d) exercise is not recommended in pregnancy
e) I don't know

21. How hard can you exercise during pregnancy?

a) only mild exercise
b) moderate exercise
c) vigorous exercise
d) until you are exhausted
e) I don't know

22. To control blood glucose effectively you should:

a) eat a healthy, balanced diet
b) do moderate exercise 5-7 days a week for about 30 minutes a day
c) spend most of your time resting
d) eat a healthy, balanced diet with moderate exercise 5-7 days a week, 30 minutes a day
e) I don't know

23. How long should you exercise per day?

a) 10 minutes
b) 15 minutes
c) till you get tired
d) 30 minutes (one 30-minute session or three 10-minute sessions)
e) I don't know

24. Should I exercise if I am overweight and unfit?

1. no, you should not
2. yes, you should start slowly and increase gradually
3. first you need to lose weight and get fit
4. I don't know

25. How can I increase my daily exercise?

a) walk children to school
b) take stairs instead of the lift or elevator
c) park the car at a distance and walk to the shopping centre
d) all of the above
e) I don't know

Management of Gestational Diabetes

26. You should check your blood glucose levels:

a) regularly for the health of you and your baby
b) occasionally
c) when you feel unwell
d) before you go to see the doctor
e) I don’t know

27. Controlling your blood glucose levels:

a) has no effect on baby
b) will give a healthy start for baby
c) has no effect on the pregnancy outcome
d) none of the above
e) I don’t know

28. If there is a social occasion, such as a party, you should:

a) not go
b) take a day off from GDM and eat whatever is served at the party
c) eat nothing during the event
d) eat before you go and take a snack
e) I don’t know

29. When your blood glucose levels are high:

a) try and work out the cause and make a note in your diary
b) just consider it to be one of those days
c) hope that tomorrow is better
d) exercise more
e) I don’t know

30. You should exercise:

a) occasionally
b) only when you feel like it
c) daily for 30 minutes
d) only when blood glucose levels are high
e) I don’t know

31. GDM can be controlled by:

a) leaving it alone
b) continuing your normal routine
c) changing to a healthy diet and exercise
d) none of the above
e) I don’t know

32. When you are hungry in between meals:

a) eat another meal
b) drink water and see if that helps
c) try and ignore it
d) go for a walk
e) I don’t know

Correct answers				
1- a,c,d	8- b	15- d	22- d	29- a
2- a,b,c	9- d	16- e	23- d	30- c
3- b	10- c	17- a	24- b	31- c
4- a,b,c	11- d	18- d	25- d	32- b
5- d	12- a	19- a	26- a	
6- c	13- b	20- c	27- b	
7- a	14- c	21- b	28- d	
