# Supplementary material for: Cross-cultural adaptation and validation to Brazilian Portuguese of the ‘knowledge of gestational diabetes (GDM)’ questionnaire for women with GDM
Source: Diabetol Metab Syndr. 2024 Sep 14;16:227. doi: 10.1186/s13098-024-01456-z (PMC11401380; doi:10.1186/s13098-024-01456-z)
Supplement: Supplementary file 2 — Additional file 2. [file 13098_2024_1456_MOESM2_ESM.pdf]

Conhecimento do Diabetes Gestacional

Abaixo estão algumas declarações sobre diabetes. Pode haver mais de uma resposta correta. Depois de ler a declaração, circule as respostas que você acredita serem verdadeiras.
Se você não souber a resposta, marque "não sei".

Essas perguntas podem ter mais de <u>uma</u> resposta correta

1. Por eu ter diabetes gestacional, meu bebê pode ser:
a. maior do que o normal
b. menor do que o normal
c. prematuro
d. admitido a unidade de tratamento intensivo
e. Não sei.

2. Como tenho diabetes gestacional, posso:
a. precisar ir ao médico com mais frequência
b. precisar de uma cesariana
c. desenvolver diabetes permanentemente no futuro
d. Não sei.

3. Se o diabetes está descontrolado, o açúcar no sangue está:
a. normal
b. elevado
c. reduzido
d. Não sei.

4. O Diabetes gestacional:
a. está presente durante a gravidez
b. Desaparece assim que o bebê nasce
c. pode levar ao diabetes no futuro
d. não é muito grave
e. Não sei.

5. O diabetes gestacional pode ser tratado com:
a. dieta
b. dieta e exercícios.
c. insulina
d. Todas as anteriores.
e. Não sei.

6. Quando meu bebê nascer:

a. Meu diabetes desaparecerá
b. Não precisarei mais me preocupar em ser diabética
c. Devo fazer um teste de glicose 6 semanas após o nascimento
d. Não sei

Conhecimento sobre o teste de nível de glicose no sangue

As perguntas a seguir exigem que você circule apenas __UM__ número.

7. Um nível normal de glicose no sangue em jejum (com o estômago vazio) é:
a. Menor que 92 mg/dL
b. Menor que 110 mg/dL
c. 130 mg/dL ou mais
d. 150 mg/dL ou mais
e. Não sei

8. Um nível normal de glicose no sangue 2 horas depois de uma refeição é:
a. Menor que 90mg/dL
b. Entre 100mg/dL e 120mg/dL
c. 130mg/dL ou mais
d. 150mg/dL ou mais
e. Não sei

9. Eu deveria testar meu nível de glicose no sangue:
a. de manhã antes do café da manhã
b. antes do almoço
c. 2 horas após as refeições
d. Alternativas a e c
e. Não sei

10. O que faço se o meu nível de glicose no sangue estiver alto?
a. anotar em meu diário de controle de glicemia
b. verificar o que comi antes do alto nível de glicose no meu sangue
c. a e b
d. Não sei.

11. O que faço se meu nível de glicose no sangue estiver alto duas vezes em uma semana?
a. anotar em meu diário de controle de glicemia
b. verificar o que eu comi antes do alto nível de glicose no sangue
c. entrar em contato com profissional da saúde de referência;
d. todas as opções acima
d. Não sei.

12. "Devo medir minha glicose no sangue se estou me sentindo mal e não comi?"
a. sim, continue medindo sua glicose no sangue como de costume
b. não, não meça sua glicose no sangue até que você esteja se sentindo melhor
c. Não sei

13. Quando você picar o dedo, você deve:

a. Usar o mesmo dedo todos os dias

b. Usar um dedo diferente a cada dia

c. Isso não é importante

d. Não sei

Conhecimento sobre sua escolha de alimentos e autocuidado após o diagnóstico de DMG

(Por favor, escolha apenas UMA opção)

14. Os tipos de alimentos com carboidratos recomendados são:

a. Pão branco

b. Pão integral

c. alimentos integrais ricos em fibras

d. alimentos ricos em amido

e. Não sei.

15. Que tipos de frutas e vegetais são melhores?

a. sucos de frutas ou de vegetais

b. frutas e vegetais processados ou enlatados

c. frutas com gorduras, açúcar e sal adicionados.

d. frutas e vegetais frescos

e. Não sei

16. Você pode ingerir proteínas a partir de:

a. carnes

b. peixes

c. nozes

d. produtos lácteos como leite ou queijo

e. todas as opções acima

f. Não sei.

17. Que tipo de proteína é melhor?

a. frango assado sem pele

b. frango com pele

c. frango frito ou salteado

d. qualquer frango

e. Não sei.

18. Uma dieta equilibrada deve ter:

a. mais vegetais

b. menos carboidratos como pão branco

c. opções com pouca gordura e pouco açúcar

d. Todas as anteriores.

e. Não sei.

19. Para o diabetes gestacional, fazer exercícios ajuda a:
a. Controlar a glicose no sangue da mãe e melhorar a saúde do bebê
b. Não ajudam
c. Cansar a mãe
d. Nenhum dos acima
e. Não sei

20. Exercícios que são recomendados durante a gravidez são:
a. Esportes de impacto, futebol, lutas, etc.
b. Correr e pular corda
c. Caminhadas, natação e ioga
d. Não são recomendados exercícios durante a gravidez
e. Não sei

21. Qual é a intensidade dos exercícios que você pode fazer durante a gravidez?
a. Apenas exercícios leves
b. Exercícios moderados
c. Exercícios vigorosos
d. Até que você esteja exausta
e. Não sei.

22. Para controlar a glicose no sangue de forma eficaz, você deve:
a. Ter uma dieta saudável e equilibrada
b. Fazer exercícios moderados 5-7 dias por semana durante cerca de 30 minutos por dia
c. Passar a maior parte do seu tempo descansando
d. Ingerir uma dieta saudável e equilibrada com 30 minutos de exercícios moderados por dia, 5-7 dias por semana
e. Não sei.

23. Por quanto tempo você deve se exercitar por dia?
a. 10 minutos
b. 15 minutos
c. até você se cansar
d. 30 minutos (uma sessão de 30 minutos ou três sessões de 10 minutos)
e. Não sei.

24. Devo me exercitar se eu estiver acima do peso e fora de forma?
a. Não, não deveria.
b. sim, você deve começar lentamente e aumentar gradualmente a intensidade
c. você precisa primeiro perder peso e ficar em forma
d. Não sei.

25. Como posso aumentar meu exercício diário?
a. Levar as crianças à escola a pé
b. Subir escadas em vez de pegar o elevador
c. Estacionar o carro a uma certa distância e caminhar até o shopping

d. Todas as anteriores.
e. Não sei.

Controle do Diabetes Gestacional

26. Você deve verificar seus níveis de glicose no sangue:
a. Regularmente para a sua saúde e a de seu bebê
b. De vez em quando
c. Quando você não se sente bem
d. Antes de ir ao médico
e. Não sei.

27. Controlar seus níveis de glicose no sangue:
a. Não afeta seu bebê
b. Permitirá um nascimento saudável para seu bebê
c. Não afeta o resultado da gravidez
d. Nenhuma das anteriores.
e. Não sei.

28. Em ocasiões sociais, como uma festa, você deve:
a. Não ir
b. Tirar um dia de folga do diabetes e comer o que for servido na festa
c. Não comer nada durante o evento
d. Comer antes de ir e levar um lanche
e. Não sei.

29. Quando os seus níveis de glicose no sangue estão elevados:
a. Tente descobrir a causa e anote em seu diário
b. Apenas o considere um "daqueles dias"
c. Espere que melhore no dia seguinte
d. Exercite-se mais
e. Não sei.

30. Você deve se exercitar:
a. De vez em quando
b. Somente quando sentir vontade
c. Diariamente por 30 minutos
d. Somente quando os níveis de glicose no sangue estão altos
e. Não sei.

31. O diabetes gestacional pode ser controlado:
a. Não dando atenção a ele
b. Continuando sua rotina normal
c. Mudando para uma dieta saudável com exercícios
d. Nenhuma dos anteriores
e. Não sei.

32. Quando você tiver fome entre as refeições:
a. Faça outra refeição
b. Beba água e veja se isso ajuda
c. Tente ignorá-la
d. Caminhe.
e. Não sei.

Respostas corretas				
1- a,c,d	1- b	15- d	22- d	29- a
2- a,b,c	2- d	16- e	23- d	30- c
3- b	3- c	17- a	24- b	31- c
4- a,b,c	4- d	18- d	25- d	32- b
5- d	5- a	19- a	26- a	
6- c	6- b	20- c	27- b	
7- a	7- c	21- b	28- d	
