# Supplementary material for: Cross-cultural adaptation and validation to Brazilian Portuguese of the ‘knowledge of gestational diabetes (GDM)’ questionnaire for women with GDM
Source: Diabetol Metab Syndr. 2024 Sep 14;16:227. doi: 10.1186/s13098-024-01456-z (PMC11401380; doi:10.1186/s13098-024-01456-z)
Supplement: Supplementary file 3 — Additional file 3. [file 13098_2024_1456_MOESM3_ESM.docx]

Supplementary Table 1: Changes made in relation to the semantic and cultural equivalency

| **Question** | **Changes made in relation to the semantic and cultural equivalency** |
| --- | --- |
| 1 | The term 'special care' was replaced by 'intensive care unit' |
| 2 | The option mentioning the regions 'India, Asia, or the Middle East' was replaced by the phrase 'are from regions where diabetes is more common’. |
| 7 | The phrase 'I should get a follow-up glucose test at my 6 weeks check-up' was replaced by 'I should have a glucose test 6 weeks after birth.' |
| 8 | Values with the unit 'mmol/L' were adapted to 'mg/dL,' as blood glucose levels are typically measured in Brazil. |
| 9 | A range of fasting glucose was added as an option to the question ('Between 100 mg/dL and 120 mg/dL'). |
| 11 | The term 'diary' was changed to 'blood glucose monitoring diary'. |
| 12 | The term 'diary' was changed to 'blood glucose monitoring diary'. |
| 18 | In the types of protein, we removed the word 'turkey' as it is not commonly consumed in Brazil, leaving only 'skinless baked chicken. |
| 28 | We replaced the phrase 'will give a healthy start for baby' with 'will allow for a healthy birth for your baby'. |
